# PM_2.5_ Emission Elemental Composition from Diverse Combustion Sources in the Metropolitan Area of Mexico City

**DOI:** 10.1100/tsw.2008.41

**Published:** 2008-03-17

**Authors:** V. Mugica, F. Mugica, M. Torres, J. Figueroa

**Affiliations:** ^1^ Universidad Autónoma Metropolitana-Azcapotzalco. Av. San Pablo No. 180, Col. Reynosa Tamaulipas Azcapotzalco, 2200 México, D.F., Mexico; ^2^ CICATA/Instituto Politécnico Nacional (IPN). Instituto Latinoamericano de la Comunicación Educativa (ILCE), Mexico

**Keywords:** source profiles, PM_2.5_, PM chemical characterization, Mexico, receptor model

## Abstract

A field study was carried out from 2003 to 2004 with the aim to develop the PM_2.5_ emission source profiles from light-duty gasoline and heavy-duty diesel vehicles, as well as emission source profiles from waste incineration, wood burning, LP gas combustion, and meat broiling. Over 25 chemical species were quantified from the fine particles emitted by the different combustion sources investigated, including organic and elemental carbon, ions, and elements. The OC/TC ratio found in the different PM_2.5_ profiles was dissimilar as well as the sulfate, nitrate, ammonium, soil species, and trace element content. Consequently, these combustion emission profiles could be used in source reconciliation studies for fine particles.

## INTRODUCTION

Mexico City's Metropolitan Area (MCMA) is one of the world's largest urban areas, with nearly 20 million inhabitants and almost 4 million vehicles within Mexico City's Basin. The large number of pollutant sources, combined with the complex topography and climate conditions of the said Basin, aggravates significantly the existing air quality problems. After ozone, the presence of particulate matter (PM) is the second largest air pollution problem, since the daily standards for STP and $PM_{10}$, are frequently exceeded[1,2]. Exposure to ambient PM pollution represents a significant health risk, particularly in major urban centers such as Mexico City[3]. Pagano et al.[4] found that finer particle-sized fractions exhibited a good correlation between mass concentration in air and mutagenic activity, and Schwartz[5] associated possible allergies and respiratory diseases with particulate air pollution.

On consideration of the Mexican expenditures on controls, calculated in several million dollars per year, and the sluggish progress toward compliance with air quality standards, a critical need exists for better and more effective abatement strategies. On the other hand, the new daily standard for $PM_{2.5}$ (65 µg $m^{-3}$) will motivate the Mexican government to identify and control emission sources contributing to ambient fine particles. In designing control strategies in other countries, several studies have been conducted to determine atmospheric concentrations of pollutants as well as emission profiles from combustion sources, since the impact of the emissions on smog formation is related to the composition of the exhaust gas[6]. Tunnel and on-road studies have been carried out in the U.S. to provide estimates of the automotive contribution to concentrations of different chemical species in the atmosphere[7,8,9]. In addition, several studies have shown that meat cooking and residential wood combustion can contribute significantly to the ambient level of fine PM due to the large amount of sites involved[10,11,12]. In other countries, PM source research has focused on the identification of chemical species, such as some elements, ions (nitrate [$NO_3^-$], sulfate [$SO_4^{2-}$], ammonium [$NH_4^+$]), total carbon (TC), organic carbon (OC) and elemental carbon (EC), to develop local PM source profiles that differentiate food cooking and wood or wood charcoal burning contributions from those coming from vehicle sources when receptor modeling source apportionment is applied[13,14,15].

Although several studies were conducted in Mexico during the last decade to determine the size distribution[16] and characterization of airborne PM, the information about the chemical composition of $PM_{2.5}$ emissions from sources is very scant. Source profiles from fugitive dust emitters were determined with a resuspension chamber[17], but most of the $PM_{2.5}$ combustion sources have not been characterized[18].

The goal of this study is to determine $PM_{2.5}$ emissions from a current on-road fleet of light-duty gasoline and heavy-duty diesel vehicles, as well as $PM_{2.5}$ emissions from waste incinerator processes, wood burning, industrial LP gas combustion, and food cooking sources. These source profiles may be applied to apportion ambient $PM_{2.5}$ concentrations to their sources by using the chemical mass receptor model (CMB) for implementing and/or designing further strategies and effective control proposals on specific sources of PM in Mexico City.

## EXPERIMENTAL METHODS

Sampling campaigns were carried out from April 2003 to January 2004. The samples for characterization of the emission profile were collected directly at the source sites during 3 days. A total of 120 samples from different sources, collected in 47-mm diameter Teflon-membrane filters (Gelman Scientific, Ann Arbor, MI), installed in calibrated Minivol samplers (Airmetrics, Eugene, OR), operating at a 5 l $min^{-1}$ flow rate, were used for mass and subsequent elemental analyses. Samples for carbon and ion analysis were collected on prefired 47-mm quartz filters (Pallflex, Products Corp., Putnam, CT). The average integrated sampling time for vehicle settings lasted 6 h in each case, and the integrated sampling time in the other sources lasted 3 h (Table 1). Background PM concentrations were measured upwind of the sources every sampling day during 8 h.

### Vehicle Emissions

Resolving contributions from diesel fuel– and gasoline-powered vehicles is a major challenge for researchers in the air quality field. Therefore, to distinguish diesel and gasoline emissions, two tunnels, one busy crossroad, one city-bus garage, and one truck station were selected for sampling PM emissions from motor vehicles in April 2003, with an average temperature of 25°C. The selected tunnels have been sampled in previous air pollution studies in Mexico City, which is why they are presently used as reference sampling places[19]. The main difference between the two tunnels used in this study is the type of vehicles crossing (light and heavy duty) as well as the neighborhoods. The first one is located near downtown on Chapultepec Avenue and is largely used by gasoline-powered vehicles with the exception of a few heavy-duty buses. It is 365 m long, 7.77 m wide, and 4.30 m high. The major vehicular traffic happens to take place during the early morning hours; thereafter, it decreases and becomes constant for the remainder of the working hours, although it picks up during peak time. Sampling equipment was installed at 2.0 m above ground level, 100 m before the exit. The second tunnel is located within an industrial area northwest of the city, in Naucalpan, and is mostly used by gasoline- and some diesel-

**TABLE 1**
**Sampling Sites**

| Source Type | Site | I.D. | Total of Samples | Description |
|---|---|---|---|---|
| Vehicle | Downtown | LDGT | 12 | Chapultepec roadway tunnel carrying light-duty gasoline vehicles (98%). |
| Vehicle | Northwest | LHDT | 12 | Naucalpan roadway tunnel carrying both light-duty gasoline vehicles and diesel trucks (90/10%). |
| Vehicle | Northwest | CRLHD | 12 | Azcapotzalco. Montevideo-San Pablo crossroad. Around 15% trucks, 15% city buses, and 70% light-duty gasoline vehicles. Located close to the Metropolitan University, industries, and a sporting club. |
| Vehicle | Northwest | BDIES | 18 | Major passenger bus terminal (Eje Central). |
| Vehicle | Northwest | TDIES | 18 | Cargo terminal, heavy-duty truck station (Naucalpan). |
| LP gas combustion | North | LPGC | 12 | Azcapotzalco, industry steam boiler. |
| Waste incineration | North | BWI | 12 | Biological waste incinerator located north of the city (Cuautitlán). |
| Waste incineration | North | MWI | 12 | Municipal waste incinerator. Metropolitan University-Azcapotzalco. |
| Meat charbroiling | North | HAMFC | 12 | Hamburger charbroiling over a charcoal grill (Cuautitlán). |
| Wood burning | North | WBUR | 12 | Pine firewood burning (Cuautitlán). |

*Note:* LDGT, light duty gasoline tunnel; LHDT, light and heavy duty tunnel; CRLHD, crossroad light and heavy duty; BDIES, bus station diesel; TDIES truck station diesel; LPGC, LP gas combustion; BWI, biological-infectious wastes incinerator; MWI, municipal wastes incinerator; HAMFC, hamburger charbroiling; WBUR, woodburning.

powered vehicles (90/10%), mainly in the morning. It is 280 m long, 10 m wide, and 9 m high, and has four one-way bores from north to south. Samplers were collocated 50 m before the exit at 2 m above ground level. One crossroad was selected because it presents heavy traffic and a different gasoline- to diesel-powered vehicles proportion. The crossroad is located at Montevideo and San Pablo Avenues, northwest of the city, right in the corner of the Metropolitan Autonomous University-Azcapotzalco campus, where 70% of the vehicles are gasoline powered and 30% are diesel vehicles. Samples were collected 1.5 m above ground level to avoid, as much as possible, resuspended particles from soil.

### *Diesel Emissions*

To develop the diesel profile, a major passenger bus terminal and one truck distribution center, also in the northern part of the city, were chosen in June 2003. The North Bus Terminal is located on Eje Central Avenue. The terminal houses several companies. Samplers were installed at 20 m before the departure exit and 1.5 m above ground level. During the 6-h sample periods, about 230 buses exited daily. Usually, the bus engines were left idling for 10 or 15 min after public announcement of each bus departure time, until all passengers were on board. The distribution center or cargo terminal is located in the manufacturing area of Naucalpan, inside a warehouse measuring approximately 200 × 100 m and 12 m high. Around 50 articulated trucks passed through the loading area during the 6-h sampling period. Samplers were installed 1.5 m above ground level and 40 m before the exit of one of the facility's two large doors. Samplings were carried out during the mornings, with temperatures around 23°C, since the afternoons were rainy.

## Meat Charbroiling, LP Gas Combustion, Wood Combustion, and Waste Incineration Emissions

Sampling campaigns during meat charbroiling, as well as during wood combustion and biological waste incineration, were carried out on the National University Campus located 32 kilometers north of Mexico City and 8 kilometers from a highway, in Cuautitlán. This place was chosen to avoid the presence of other sources such as vehicles and industries. Because these sampling campaigns were not carried out using a dilution chamber, this study has limitations and emission rates could not be estimated. To avoid direction changes of the emitted pollutant plumes, a fan was used to address the smoke to the samplers during the 3-h sampling periods.

Samples of municipal waste incineration were taken at another University campus located in Azcapotzalco, northwest of Mexico City. Case samples were taken isokinetically in the chimney's sampling port, as well as downstream on a roof located 6 m from the chimney.

### *Meat Charbroiling*

The chemical composition of particles emitted from meat charbroiling was determined in October 2003, from samples collected at a restaurant currently doing business and located in a low-traffic street in the town of Cuautitlán. The restaurant has a charcoal grill for hamburger and meat cooking, and has an area of 40 $m^2$. The cooking smoke in the facility was withdrawn through an overhead exhaust hood. The samplers were placed on the roof of the facility. Each sample collection was carried out during 3 h, downstream of the ventilation systems servicing the charbroiling grill. The temperature at the sampling site was about 24°C.

### *Wood Combustion*

Pine woods were used to characterize particulate emissions from wood burning, not only because wood is used for residential heating, but also because wild fires are common during the dry season, contributing to the presence of airborne particles. Around 20 kg of dry branches of pine firewood were burned using a roasting spit. A fan was used to address the smoke to the samplers located at a distance of 3 and 5 m.

### *Biological-Infectious Wastes Incinerator (BWI)*

This incinerator was designed for burning the agricultural, food processing, veterinarian, and clinic wastes from the different colleges of the University. This facility usually works two times a week; the incinerator uses diesel as fuel, has a single combustion chamber, and is located in a closed room of 3 × 3 m and 3 m high. The sampling campaign was performed in November 2003 and samplers were located on the roof at a distance of 5 m of the stack. Ambient temperature during the sampling period was around 26°C. The incinerator is not equipped with a dust collector or gas-control apparatus.

### *Municipal Wastes Incinerator (MWI)*

The Goodrich incinerator, with two combustion chambers, is located on the Azcapotzalco campus of the Metropolitan University. Around 50 kg of wastes from the campus were burned each day, using LP gas as fuel. Samples were taken in January 2004. Ambient temperature during the sampling period was around 26°C. The incinerator is not equipped with a dust collector or gas-control apparatus.

### *Industrial Steam Boiler*

The LP gas steam boiler, with 1 $m^3$ of capacity, is located in an industrial area of Azcapotzalco. Samples were taken in January 2004 and samplers were located on the roof 7 m downwind of the stack during 12 h. Ambient temperature during the sampling period was around 26°C. The boiler is not equipped with a dust collector or gas-control apparatus.

## Chemical Analysis

All Teflon filters were weighed before and after sample collection in a Mettler Toledo ultra balance. The filters were conditioned in a chamber maintained at RH = 40 ± 5% and T = 20 ± 2°C before and after sampling and weighing. The weighed filters were stored in a freezer until aerosol sampling and chemical analyses took place. Inductively Coupled Plasma-Atomic Emission Spectrometry (ICP AES, Atom Advantage Thermo Jarrel Ash) was used to analyze the elemental components of the $PM_{2.5}$ collected on the filters. Prior to the ICP analysis, each Teflon filter was digested in a microwave oven (OI-Analytical) using high-pressure Teflon digestion vessels with 2 ml of HF, 1 ml HCl, and 2 ml $HNO_3$ (67%). Average filter blank value was used as a background subtraction for each sampled filter. Elemental composition was determined also by X-ray fluorescence (XRF, Siemens) Quartz-fiber filters were fractioned to quantify OC and EC, and for water-soluble ions. Soluble ions were extracted ultrasonically (Branson bath) with Milli-Q deionized water during 20 min. Sulfate ($SO_4^{2-}$), water-soluble ammonium ($NH_4^+$), nitrate ($NO_3^-$), water-soluble sodium ($Na^+$), and potassium ($K^+$) were quantified by ion chromatography (Perkin Elmer-Alltech 550 with conductivity detector), using specific anion and cation Alltech columns. OC and EC were determined by an automated thermal-optical transmittance (TOT) carbon analyzer (Sunset Lab), using method 5040 protocol (NIOSH)[20].

## Quality Assurance

The quartz-fiber filters were baked during 12 h at 500°C prior to sampling to reduce residual carbon levels associated with new filters. Duplicate samples of Teflon and quartz filters were collected at each site every day. Samples were analyzed in triplicate and certified standards were used. Extractions of 20 mg of a well-characterized urban dust (SRM 1649a standard reference material NIST), field samples, and filter blanks were handled and analyzed under the same procedure. Intercomparison and performance tests were carried out between CICATA-Altamira and UAM-Azcapotzalco. For the purposes of calculating weight fractions, elements were normalized for oxygenated species as described by Mc Donald[13].

## RESULTS AND DISCUSSION

Table 2 shows the average $PM_{2.5}$ concentrations measured in the sampling sites. The highest on-road concentrations were found in the Naucalpan tunnel, whereas the measured emissions into the cargo station and the bus terminal were 161.34 and 198.35 µg $m^{-3}$, respectively. These high values could represent a health risk for exposed workers during their 8-h work shifts within the stations. On the other hand, Table 3 shows the $PM_{2.5}$ background measurements carried on upwind of the incineration, wood burning, and charbroiling processes; in all cases, ambient concentrations were below the proposed 24-h standard of 65 μg $m^{-3}$.

**TABLE 2**

**$PM_{2.5}$ Concentrations at Vehicle Emission Sampling Sites**

| Site | Source | Date, 2003 | Average Measured Concentrations (μg $m^{-3}$) |
|---|---|---|---|
| Chapultepec tunnel | Gasoline vehicles | April | 175.23 ± 38.47 |
| Naucalpan tunnel | Diesel and gasoline vehicles | April | 193.31 ± 37.16 |
| Azcapotzalco crossroad | Diesel and gasoline vehicles | April | 99.27 ± 32.23 |
| North Bus Terminal | Buses (diesel) | June | 198.35 ± 27.89 |
| Cargo station, Naucalpan | Trucks (diesel) | June | 161.34 ± 43.66 |

**TABLE 3**
**Background Upwind Concentrations during Emissions Source Sampling**

| Site | Source | Date | Background Concentrations (μg $m^{-3}$) |
|---|---|---|---|
| Cuautitlán UNAM | Charbroiling | October 2003 | 32.57 ± 9.51 |
| Cuautitlán UNAM | Wood burning | October 2003 | 26.42 ± 11.67 |
| Cuautitlán UNAM | BWI | November 2003 | 36.93 ± 10.39 |
| Azcapotzalco | MWI and LPGC | January 2004 | 54.15 ± 18.93 |

Table 4 presents the eight developed combustion $PM_{2.5}$ source profiles as mass fractions of chemical species, which were in detectable concentrations. The abundance of each specie in the source profile is the ratio of each concentration (in μg $m^{-3}$) to the total mass (in μg $m^{-3}$). Although the concentrations in the truck station and the bus garage were different, the proportions of chemical species were quite similar; therefore, only one profile was constructed to represent diesel-powered vehicle emissions (DIESEL). It is important to mention that the elements' concentrations measured were quite comparable for both methods, ICP and XRF. However, generally, ICP results were a little higher, perhaps due to the break up of fine particles, which can liberate some elements from within them. As the differences between the two methods were less than 6%, the percent mass reported is the average of all results obtained for each element.

$PM_{2.5}$ was mostly carbonaceous material from 55 to 88%, and the largest part of this carbon emission was classified as organic. In comparison with fugitive dust profiles determined for Mexico City[17], where geological material (Ca, Fe, Al, and Si) was the major component, combustion profiles presented mostly carbonaceous material and enough differences in the content of trace elements (Fig. 1). Therefore, the profiles determined in this study can be used in combination with dust profiles for source reconciliation estimations, not only in Mexico City, but also in other Mexican or Latin American cities where the same kind of fuels or wastes are combusted.

## Vehicle Profile Results

As shown in Table 4 and Fig. 2, the percent content of chemical species is different for the four types of vehicle profiles. The measured TC varied from 55% for the light-duty vehicle profile to 79% in the diesel profile**,** which also has the highest content of EC (35%). The EC content from diesel emissions is similar to that found in other studies, although the OC content was an average 40% higher in our study[9,21]. The crossroad profile also has a high content of TC, but it could be influenced by other sources, such as vegetal material from the University gardens, emissions from a large pastry factory located two blocks away from the University, or other industries near the site.

The different OC/TC ratios for the $PM_{2.5}$ vehicle profiles suggest that this fact can be due to differences in the vehicular fleet, since this ratio is around 10% higher for light-duty vehicles than for heavy-duty diesel vehicles. The OC/TC ratios in the profiles developed where both diesel- and gasoline-powered vehicles traveled were quite similar for the Naucalpan tunnel (0.64) and crossroad (0.65). The diesel vehicle profile presented the lowest OC/TC ratio (0.59), whereas the light-duty vehicle profile had the highest (0.66).

Contents of soil species such as Al, Si, Ca, and Fe are present in the four vehicle profiles to compose 4–8% of the mass. The high content of soil components in the Naucalpan tunnel can be due to the large extensions of eroded soils close to it. The percentage of cations and anions is quite similar in vehicle profiles (around 5 and 15%, respectively) except for the Insurgentes tunnel (almost only gasoline-powered vehicles), which reported lower abundance, 4% of cations and 11% of anions. Trace species represented around 4%.

**TABLE 4**
**Developed $PM_{2.5}$ Combustion Source Profiles (% mass)**

| | LDGT | LHDT | CRLHD | DIESEL | LPGC | BWI | MWI | WBUR | HAMFC |
|---|---|---|---|---|---|---|---|---|---|
| $Cl^-$ | 0.23 ± 0.05 | 1.77 ± 0.19 | 0.23 ± 0.03 | 1.06 ± 0.21 | 0.67 ± 0.00 | 0.15 ± 0.07 | 6.45 ± 1.28 | 0.20 ± 0.03 | 0.78 ± 0.12 |
| $NO_3^-$ | 5.01 ± 1.77 | 6.83 ± 1.04 | 8.03 ± 1.04 | 6.04 ± 3.03 | 0.00 ± 0.00 | 0.32 ± 0.19 | 0.15 ± 0.03 | 0.19 ± 0.03 | 0.59 ± 0.13 |
| $SO_4^{2-}$ | 5.64 ± 1.17 | 6.93 ± 1.31 | 7.47 ± 1.21 | 7.43 ± 1.89 | 0.00 ± 0.00 | 0.19 ± 0.11 | 1.35 ± 0.27 | 0.42 ± 0.07 | 0.80 ± 0.14 |
| $NH_4^+$ | 2.73 ± 0.54 | 4.66 ± 0.64 | 4.60 ± 0.66 | 4.35 ± 0.85 | 0.00 ± 0.00 | 0.09 ± 0.04 | 0.25 ± 0.06 | 0.12 ± 0.01 | 0.23 ± 0.03 |
| $Na^+$ | 0.56 ± 0.19 | 0.47 ± 0.08 | 0.35 ± 0.09 | 0.16 ± 0.01 | 0.01 ± 0.00 | 0.09 ± 0.03 | 2.80 ± 0.55 | 0.03 ± 0.01 | 0.47 ± 0.11 |
| $K^+$ | 0.44 ± 0.07 | 0.52 ± 0.02 | 0.54 ± 0.06 | 0.37 ± 0.09 | 0.02 ± 0.00 | 0.48 ± 0.28 | 2.94 ± 0.35 | 0.57 ± 0.08 | 0.48 ± 0.04 |
| OC | 38.04 ± 7.68 | 42.22 ± 5.44 | 43.34 ± 2.12 | 40.06 ± 7.76 | 71.32 ± 5.04 | 69.18 ± 7.47 | 64.28 ± 11.36 | 74.58 ± 10.19 | 74.19 ± 12.20 |
| EC | 16.57 ± 3.10 | 22.01 ± 3.73 | 23.20 ± 4.15 | 32.34 ± 6.94 | 5.353 ± 0.35 | 1.15 ± 0.96 | 6.98 ± 1.26 | 13.13 ± 2.76 | 3.06 ± 0.92 |
| Mg | 0.32 ± 0.06 | 0.10 ± 0.01 | 0.10 ± 0.02 | 0.06 ± 0.01 | 0.00 ± 0.00 | 0.01 ± 0.00 | 0.98 ± 0.13 | 0.10 ± 0.01 | 0.01 ± 0.00 |
| Al | 0.33 ± 0.05 | 0.73 ± 0.13 | 0.67 ± 0.08 | 0.88 ± 0.13 | 0.00 ± 0.00 | 0.00 ± 0.00 | 0.36 ± 0.08 | 0.03 ± 0.00 | 0.03 ± 0.00 |
| Si | 1.60 ± 0.31 | 2.91 ± 0.05 | 2.21 ± 0.09 | 2.64 ± 0.58 | 0.02 ± 0.00 | 0.01 ± 0.00 | 0.11 ± 0.03 | 0.03 ± 0.02 | 0.06 ± 0.02 |
| Ph | 0.07 ± 0.02 | 0.00 ± 0.00 | 0.06 ± 0.01 | 0.03 ± 0.01 | 0.01 ± 0.00 | 0.02 ± 0.01 | 0.01 ± 0.00 | 0.01 ± 0.00 | 0.05 ± 0.01 |
| S | 1.20 ± 0.31 | 1.52 ± 0.27 | 2.59 ± 0.13 | 2.35 ± 0.42 | 0.00 ± 0.00 | 0.02 ± 0.01 | 0.00 ± 0.00 | 0.18 ± 0.02 | 0.33 ± 0.05 |
| Ca | 0.88 ± 0.22 | 2.64 ± 0.03 | 0.52 ± 0.06 | 0.83 ± 0.17 | 0. 92 ± 0.032 | 0.95 ± 0.36 | 3.26 ± 0.63 | 0.05 ± 0.02 | 0.03 ± 0.01 |
| Ti | 0.04 ± 0.01 | 0.43 ± 0.05 | 0.07 ± 0.02 | 0.19 ± 0.04 | 0.00 ± 0.00 | 0.00 ± 0.00 | 0.03 ± 0.00 | 0.01 ± 0.00 | 0.00 ± 0.00 |
| V | 0.02 ± 0.01 | 0.05 ± 0.01 | 0.03 ± 0.00 | 0.09 ± 0.02 | 0.00 ± 0.00 | 0.00 ± 0.00 | 0.02 ± 0.03 | 0.00 ± 0.00 | 0.00 ± 0.00 |
| Cr | 0.01 ± 0.01 | 0.01 ± 0.00 | 0.01 ± 0.00 | 0.00 ± 0.00 | 0.01 ± 0.00 | 0.00 ± 0.00 | 0.01 ± 0.01 | 0.00 ± 0.00 | 0.00 ± 0.00 |
| Mn | 0.05 ± 0.04 | 0.05 ± 0.01 | 0.08 ± 0.01 | 0.02 ± 0.01 | 0.00 ± 0.00 | 0.00 ± 0.00 | 0.02 ± 0.00 | 0.00 ± 0.00 | 0.00 ± 0.00 |
| Fe | 1.59 ± 0.22 | 1.72 ± 0.05 | 0.83 ± 0.11 | 0.49 ± 0.07 | 0.00 ± 0.00 | 0.00 ± 0.00 | 0.62 ± 0.12 | 0.02 ± 0.01 | 0.01 ± 0.00 |
| Co | 0.01 ± 0.00 | 0.00 ± 0.00 | 0.00 ± 0.00 | 0.00 ± 0.00 | 0.00 ± 0.00 | 0.00 ± 0.00 | 0.00 ± 0.00 | 0.00 ± 0.00 | 0.00 ± 0.00 |
| Ni | 0.01 ± 0.00 | 0.02 ± 0.01 | 0.01 ± 0.00 | 0.01 ± 0.00 | 0.00 ± 0.00 | 0.00 ± 0.00 | 0.23 ± 0.04 | 0.01 ± 0.00 | 0.00 ± 0.00 |
| Cu | 0.11 ± 0.03 | 0.06 ± 0.02 | 0.14 ± 0.04 | 0.05 ± 0.01 | 0.01 ± 0.00 | 0.00 ± 0.00 | 0.11 ± 0.02 | 0.01 ± 0.00 | 0.00 ± 0.00 |
| Zn | 0.33 ± 0.09 | 0.30 ± 0.03 | 0.17 ± 0.04 | 0.23 ± 0.04 | 0.00 ± 0.00 | 0.00 ± 0.00 | 0.31 ± 0.08 | 0.00 ± 0.00 | 0.01 ± 0.00 |
| Cd | 0.00 ± 0.00 | 0.01 ± 0.00 | 0.02 ± 0.01 | 0.02 ± 0.01 | 0.00 ± 0.00 | 0.00 ± 0.00 | 0.04 ± 0.00 | 0.00 ± 0.00 | 0.00 ± 0.00 |
| Sn | 0.01 ± 0.01 | 0.03 ± 0.01 | 0.02 ± 0.01 | 0.03 ± 0.00 | 0.00 ± 0.00 | 0.00 ± 0.00 | 0.02 ± 0.01 | 0.00 ± 0.00 | 0.00 ± 0.00 |
| Ba | 0.18 ± 0.06 | 0.19 ± 0.05 | 0.10 ± 0.03 | 0.10 ± 0.03 | 0.01 ± 0.00 | 0.00 ± 0.00 | 0.01 ± 0.00 | 0.01 ± 0.00 | 0.01 ± 0.00 |
| Hg | 0.00 ± 0.00 | 0.00 ± 0.00 | 0.00 ± 0.00 | 0.00 ± 0.00 | 0.00 ± 0.00 | 0.00 ± 0.00 | 0.03 ± 0.00 | 0.00 ± 0.00 | 0.00 ± 0.00 |
| Pb | 0.16 ± 0.05 | 0.19 ± 0.02 | 0.13 ± 0.05 | 0.11 ± 0.03 | 0.00 ± 0.00 | 0.00 ± 0.00 | 0.02 ± 0.01 | 0.00 ± 0.00 | 0.01 ± 0.00 |
| OC/TC | 0.66 | 0.64 | 0.65 | 0.59 | 0.93 | 0.98 | 0.90 | 0.85 | 0.96 |

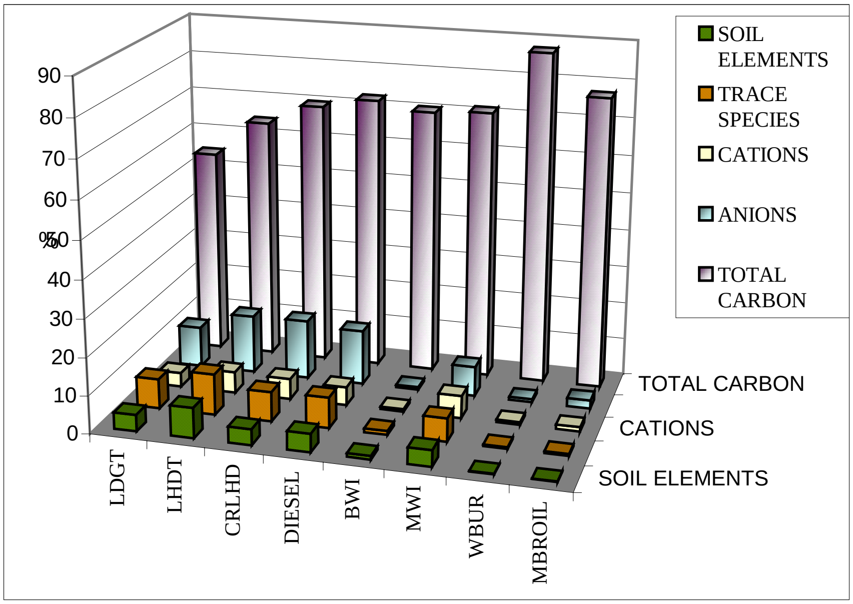

**FIGURE 1.** Comparison of $PM_{2.5}$ emission profiles. Total carbon (OC, EC), soil elements (Si, Al, Fe, Ca), trace elements (Ph, S, Ca, Ti, V, Cr, Mn, Fe, Co, Ni, Cu, Zn, Cd, Sn, Ba, Hg, Pb), anions ($Cl^-$, $SO_4^{2-}$, $NO_3^-$), cations ($Na^+$, $K^+$, $NH_4^+$).

## Incineration and Combustion Process Profiles

The contents of carbonaceous material from both incineration processes were alike, although OC/TC ratio was higher for BWI (0.98) as compared with MWI (0.90). The contents of other species were quite different in both profiles**,** as can be appreciated in Fig. 3. With the exception of $NO_3^-$, the ion content was higher in MWI than in BWI (14 and 1%, respectively); especially $Cl^-$, which reached average 6% for BWI. The $Cl^-$ content in this emission source is the highest of all the developed profiles. Soil species (Al, Si, and Fe) are present in MWI emissions, but not in BWI emissions. Such a fact also makes this last profile different from all others. The analysis reveals that MWI produces detectable concentrations of trace metals such as Ni, Cu, Zn, Cd, Sn, Pb, and Hg, which were not detectable in the rest of the profiles. The presence of these species as well as $Cl^-$ is due to bad separation of municipal wastes that have some metal containers, pieces of plastics, or synthetic fibers, etc. Emissions from the LP gas boiler presented an OC/EC ratio of 0.88, similar to municipal incineration.

## Wood Burning Profile

TC content was 88% with an OC/TC ratio of 0.85. Potassium was the most abundant element after carbon content, its percentage being the highest in comparison to all other sources (0.6%). Smaller quantities of $SO_4^{2-}$, $NO_3^-$, $Cl^-$, $NH_4^+$, and Na were also present in wood burning emissions. These trace elements have been commonly used with the total OC and EC concentration to apportion wood smoke in studies that did not utilize organic chemical components[13]. Soil species were detected as well as some trace elements, such as Ti, Zn, Ni, and Ba.

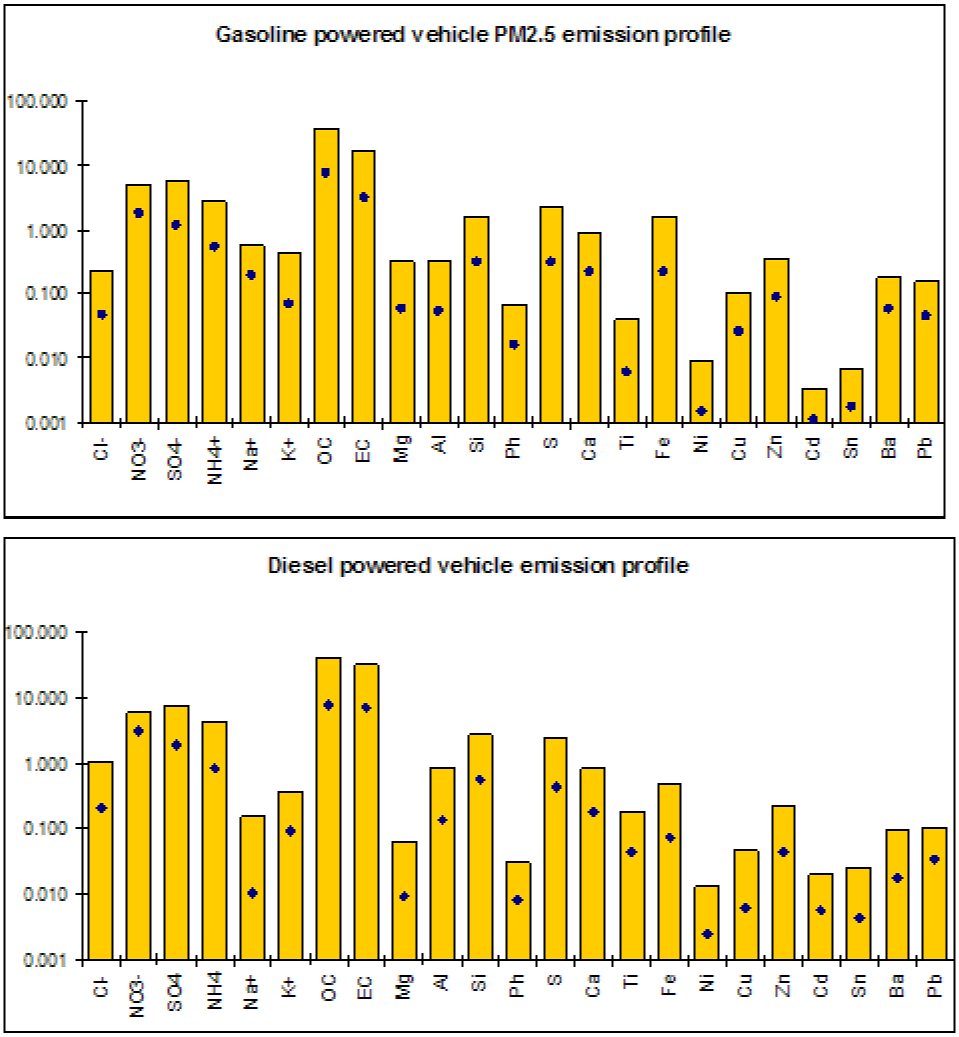

**FIGURE 2.** Comparison of gasoline- and diesel-powered vehicle $PM_{2.5}$ emission profiles. Dots represent uncertainties.

## Meat Charbroiling Profile

More than 30,000 places where food is cooked are registered in Mexico City, and more than 3,000 small informal places that prepare all kinds of meals can be found on the City's sidewalks. These places use LP gas as well as charcoal or firewood. Frequently, large smoke plumes can be observed coming out from those sites; although some restaurants have chimneys to avoid disturbing the consumers, there are no control mechanisms to reduce the emissions from these sources to the atmosphere. The composition distribution of particles collected from meat cooking shows that the $PM_{2.5}$ emitted is almost 75% OC and has a very small EC content. The 75% OC is a higher percentage than that found in other studies[10]. All ionic species were found in meat smoke with abundances of 0.80, 0.78, and 0.59% for $SO_4^{2-}$, $Cl^-$, and $NO_3^-$, respectively. Content of inorganic species was very small except for K, Na, and S. Smaller quantities of Al, Si, P, Ca, Mg, and Fe were also detected in the fine PM emitted from meat charbroiling.

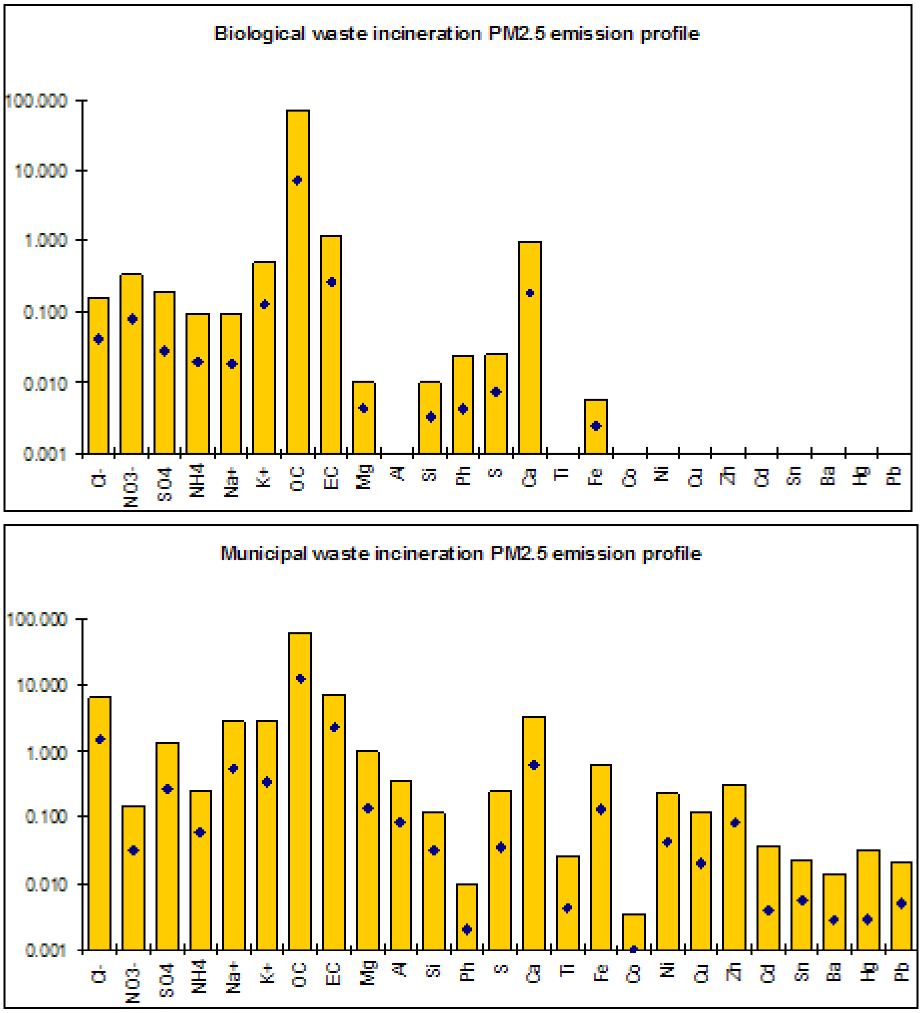

**FIGURE 3**. Comparison of BWI and MWI $PM_{2.5}$ emission profiles. Dots represent uncertainties.

## CONCLUSIONS

Despite the limitations of this study, since rate emissions could not be estimated, eight distinct composite emission profiles were constructed to represent light-duty vehicles, combination of light- and heavy-duty vehicles, heavy-duty diesel vehicles, biological waste incineration, municipal waste incineration, firewood combustion, LP gas combustion, and meat charbroiling. The profiles developed showed clear differences in chemical composition among them. Vehicle profiles showed that upon increasing emissions from powered diesel vehicles, the EC contents also increase**.** The highest OC/TC ratio was found for biological waste incineration emissions followed by meat charbroiling, then LP gas combustion. The presence of some trace metals and $Cl^-$ distinguishes municipal waste incineration profiles, whereas wood burning exhibited the greatest K content. Ions fraction is useful also to ascribe fine particle emissions to combustion sources.

Carbon and other chemical species contents are quite different as compared with fugitive dust emission profiles from paved, unpaved, and agricultural soils determined for Mexico City in preceding studies; consequently, the source profiles determined in this study could be applied to apportion ambient $PM_{2.5}$ concentrations to their sources by using the chemical mass receptor model (CMB), which is indeed useful for further proposals of control strategies on specific sources of particulate matter in Mexico City.

## ACKNOWLEDGMENTS

The authors wish to express their thanks for the chemical analysis to the Applied Chemistry laboratories at the Metropolitan University-Azcapotzalco, CICATA/IPN, and Intense S.A. Some supplies were acquired through project FIES-96-04-VI supported by IMP and PEMEX. V. Mugica gratefully acknowledges the SNI for the distinction of her membership and the stipend received.
